# Uncertainty-Calibrated Safety Gating for Vision–Language– Action Manipulation Under Domain Shift: Reliability Gains and Intervention–Efficiency Trade-Offs

**DOI:** 10.3390/s26103140

**Published:** 2026-05-15

**Authors:** Atef M. Ghaleb, Ali S. Allahloh, Sobhi Mejjaouli, Mohammed A. H. Ali, Adel Al-Shayea

**Affiliations:** 1 Department of Industrial Engineering, College of Engineering and Advanced Computing, Alfaisal University, Riyadh 11533, Saudi Arabia; aghaleb@alfaisal.edu (A.M.G.);; 2 Department of Electrical Engineering, Zakir Husain College of Engineering and Technology (ZHCET), Aligarh Muslim University, Aligarh 202002, India; 3 Department of Mechanical Engineering, Faculty of Engineering, Universiti Malaya, Kuala Lumpur 50603, Malaysia; 4 Industrial Engineering Department, College of Engineering, King Saud University, Riyadh 115421, Saudi Arabia

**Keywords:** autonomous robots, embodied intelligence, vision–language–action, uncertainty calibration, runtime assurance, multimodal sensing, domain shift, safety-critical autonomy, robot manipulation, robust decision-making

## Abstract

Vision–Language–Action (VLA) policies promise flexible long-horizon manipulation, but deployment under domain shift requires both reliable uncertainty estimates and a workable runtime-assurance policy. We study a model-agnostic uncertainty-calibrated safety-gating wrapper that estimates online failure risk and routes control among policy execution, pause-and-reobserve, and a fallback planner. Using a cleaned and consistently aggregated benchmark pipeline, we evaluate two long-horizon manipulation tasks in NVIDIA Isaac Sim 5.0 under lighting, texture, occlusion, sensor, and combined shifts. Relative to an ungated VLA baseline, calibrated gating improves mean shifted success from 57.5% to 77.2% and reduces aggregate expected calibration error from 0.303 to 0.100. The largest success gains occur under occlusion and combined shift, including improvements from 48.3% to 85.2% on the drawer task and from 59.4% to 87.8% on clutter sort. The results also expose a systems trade-off: an aggressive uncalibrated threshold baseline attains stronger raw success and collision metrics, but requires nearly twice as many interventions per shifted episode (21.6 vs. 11.5). The main contribution is, therefore, an empirical characterization of the reliability–intervention trade-off created by calibrated supervision, not a claim that the calibrated supervisor is universally the best terminal controller. We frame calibrated gating as a better-calibrated, lower-intervention supervisor that materially improves robustness relative to an ungated VLA while revealing the open problem of mapping calibrated risk into efficient intervention policies. Additional threshold-sensitivity, signal-diagnostic, overhead, and residual-failure analyses show that the selected operating point is meaningful but not universal: the calibrated risk threshold captures most shifted failures in retrospective logs, yet residual contacts still arise during pause and fallback states. These findings provide controlled simulation evidence for trustworthy VLA supervision under distribution shift and clarify the reliability–intervention frontier that future embodied-control systems must navigate.

## 1. Introduction

Recent advances in multimodal foundation models have produced vision–language–action (VLA) policies that can interpret visual observations and language instructions to directly output continuous robot actions. Pioneered by works like RT-2 [1], PaLM-E [2], and OpenVLA [3], these large-scale policies have demonstrated impressive generalization across tasks and embodiments. However, deploying such end-to-end policies in the real world remains challenging: models often exhibit overconfidence in novel scenarios and can produce unstable or unsafe motions under distribution shifts. In safety-critical manipulation—e.g., a robot retrieving objects from clutter or operating near humans—blindly trusting an uncalibrated policy can lead to collisions or task failure.

In this work, we address the problem of safe long-horizon robotic manipulation under domain shift by introducing a novel uncertainty-calibrated safety-gating mechanism. Our method acts as a meta-controller that monitors the VLA policy’s confidence at runtime and decides when to proceed with the learned policy vs. when to intervene (slow down, re-sense, or switch to a proven safe fallback controller). By wrapping the policy rather than modifying its internals, our approach is model-agnostic and can work with any VLA policy that provides an estimate of its output uncertainty.

The core insight is to exploit the rich information in a modern policy’s output distribution (or an ensemble of policies) to predict the risk of error. We calibrate this predictive uncertainty using held-out validation scenarios so that the gating threshold corresponds to a concrete failure probability (e.g., intervene if predicted success < 80%). When the threshold is breached, the system triggers a predefined fallback strategy implemented via classical motion planning (MoveIt 2) to ensure safety. This integration of learned and classical controllers yields a form of hybrid autonomy: the robot attempts tasks with an agile learned policy when confident, but reverts to cautious, verified maneuvers when uncertain, akin to a self-driving car handing control to an ADAS system in adverse conditions.

We validate our approach on two complex, multi-step manipulation tasks: (i) opening a drawer, picking a target object inside, and placing it into a bin; and (ii) a cluttered tabletop sort-and-place task requiring re-grasps. Using NVIDIA Isaac Sim 5.0, we sweep structured lighting, material, occlusion, sensor, and combined shifts and compare four methods: the base VLA policy, an uncalibrated uncertainty-threshold baseline, our calibrated gating policy, and a fallback-on-failure hybrid. The cleaned benchmark package shows a nuanced but practically informative picture. Calibrated gating consistently improves shifted success relative to the ungated policy and substantially improves predictive calibration under shift. However, the strongest raw benchmark success and collision numbers are achieved by a more aggressive uncalibrated threshold baseline that intervenes far more frequently. Consequently, the main contribution of the paper is the empirical characterization of a calibrated supervision operating point on the reliability–intervention frontier: calibrated gating is more interpretable and less intervention-heavy than the aggressive threshold controller, while remaining substantially more robust than no supervision. In response to the operating-point questions raised by the benchmark, we additionally report threshold sensitivity, condition-wise calibration transfer, uncertainty-signal diagnostics, fallback/residual-failure analysis, and cumulative runtime overhead.

### 1.1. Main Contribution and Scope

The paper should be read as an empirical runtime-supervision study rather than as a new base VLA model or a proof of physical-robot safety. Its primary contribution is to quantify how post hoc calibration changes the operating point of a VLA supervisor under controlled domain shift, including the price paid in interventions and runtime. All quantitative claims are bounded to the cleaned Isaac Sim benchmark described below; no matched real-robot validation is claimed.

### 1.2. Contributions

Key contributions of this work include:

- **A model-agnostic calibrated runtime supervisor:** We present an uncertainty-calibrated safety-gating wrapper for VLA manipulation that estimates failure risk online and maps it to explicit intervention modes (proceed, pause/reobserve, fallback), without retraining the underlying foundation policy for safety.
- **Hybrid policy integration with explicit intervention accounting:** We combine learned VLA control with a classical MoveIt 2 fallback planner and treat intervention burden as a first-class systems metric rather than an implementation detail.
- **Structured domain-shift evaluation:** We provide a controlled Isaac Sim benchmark spanning lighting, material, occlusion, sensor, and combined shifts, all regenerated under a single cleaned aggregation rule.
- **Evidence-backed operating-point analysis:** The cleaned results show that calibrated gating improves shifted robustness and predictive reliability relative to an ungated VLA (aggregate ECE 0.303→0.100, NLL 0.670→0.485), while also exposing a reliability–intervention frontier: a more aggressive uncalibrated threshold baseline attains stronger raw benchmark metrics at the cost of roughly 2× more interventions per shifted episode.
- **Diagnostic analyses of reviewer-critical factors:** We explicitly examine threshold sensitivity, calibration behavior beyond the calibration split, component-level uncertainty signals, fallback/planner residual failures, cumulative overhead, and failure phases so that the practical limits of the method are visible rather than implicit.

## 2. Related Work

### 2.1. Vision–Language–Action (VLA) Policies for Robotics

Foundation models that couple vision and language with action outputs have recently enabled robots to perform diverse tasks specified in natural language. **Robotics Transformer 2 (RT-2)** by Google DeepMind established this paradigm, fine-tuning a vision–language model on robotic data to directly output tokenized actions. RT-2 demonstrated zero-shot generalization to novel objects and instructions by leveraging web-scale vision–language pretraining. Subsequently, **PaLM-E** integrated a large language model with embodied sensor inputs, achieving impressive multi-task performance and even reasoning via chain-of-thought in a single model. Open-source efforts include **OpenVLA** [3], a 7B-parameter VLA model trained on 970 k real robot episodes, and **Octo** [4], a 93 M-parameter transformer policy trained on the Open X-Embodiment dataset. These models (and others like RT-1 [5], VC-1, etc.) form a new class of generalist robot policies that can be adapted to various platforms and tasks.

At the same time, recent benchmarking work has made clear that VLA competence remains fragile when conditions move away from curated training settings. **VLATest** introduced fuzzing-style evaluation for robotic manipulation and reported substantial robustness gaps across representative VLA systems [6]. **Eva-VLA** further showed that object transformations, illumination changes, and adversarial visual variations can drive severe degradation in long-horizon manipulation performance [7]. More recently, **FPC-VLA** added an explicit supervisor for failure prediction and correction, demonstrating the growing importance of runtime oversight rather than pure end-to-end execution [8]. Our work is complementary to these directions. We do not introduce a new base VLA or a learned correction head; instead, we study how a lightweight, post hoc calibrated supervisor can monitor a pre-trained policy, improve reliability under shift, and expose the practical trade-off between raw benchmark performance and intervention burden. This is also related in spirit to *SayCan* [9], where a high-level language model defers to a value function to decide if an instruction is feasible. In contrast, we target low-level control decisions, using calibrated uncertainty estimates as the intervention signal.

### 2.2. Uncertainty Estimation and Calibration in Robot Policies

Deep neural policies are known to be poorly calibrated, often outputting high-confidence predictions even when wrong. For safe robotics, it is crucial to quantify a model’s *epistemic uncertainty* (due to limited knowledge) versus *aleatoric uncertainty* (due to inherent noise). Techniques like **Deep Ensembles** [10] and **Monte Carlo (MC) Dropout** [11] have been applied to estimate uncertainty in perception and control. Kendall and Gal [12] introduced a framework to obtain both epistemic and aleatoric uncertainties from deep vision models, laying groundwork for many robotics applications. In reinforcement learning, **Ensemble Bootstrapping** (e.g., Bootstrapped DQN) and distributional methods (Quantile Regression DQN) have been combined to detect novel states. Notably, Hoel et al. [13] proposed **Ensemble Quantile Networks (EQNs)** for autonomous driving, using an ensemble of distributional *Q*-networks to quantify total uncertainty and trigger a safe policy when uncertainty was above a threshold. They showed that this approach can avoid dangerous decisions in unseen road situations.

Beyond estimation, *calibration* is critical so that the model’s confidence corresponds to true success likelihood. Guo et al. [14] demonstrated that modern networks are often miscalibrated and popularized **temperature scaling** as a simple post hoc fix. Calibration of robotic policies has gained renewed attention in the VLA era. Zollo and Zemel presented one of the first systematic studies of confidence calibration specifically for VLA models and showed that lightweight post hoc calibration can materially improve reliability without changing the base controller [15]. Valle et al. argued that binary task success is insufficient on its own and proposed VLA-specific uncertainty and execution-quality metrics that correlate with expert judgments [16]. Wu et al. [17] explicitly calibrated a language-conditioned policy’s confidence using a small demonstration set, and then proposed an uncertainty-aware action selection strategy. In their experiments on manipulation benchmarks, calibration plus a modified action selection improved success rates without fine-tuning the model. Our work is aligned with this trend, using temperature scaling (and comparing alternatives like isotonic regression) to calibrate a policy’s predicted success probabilities. We extend beyond pure action selection to an integrated gating mechanism that can override the policy entirely, and we explicitly measure the resulting success–safety–intervention trade-off in closed-loop control.

### 2.3. Safe Learning and Policy Gating

Ensuring safety in learning-based control has been studied in safe reinforcement learning and motion planning. A comprehensive overview is provided by Garcia and Fernández [18]. Classical approaches include **hard constraints** or **shielding**—e.g., using control barrier functions or verifying a candidate action against a safety model before execution. In deep RL, **risk-sensitive criteria** like CVaR (Conditional Value-at-Risk) have been introduced to bias policies against low-probability catastrophic outcomes, and safe RL algorithms enforce chance constraints on expected cost. However, many of these methods require shaping the training process; by contrast, a *runtime gating* strategy like ours can be retrofitted to a policy after training, which is advantageous for large foundation models.

Our approach falls under **mediator architectures**: a supervisory layer that monitors a learned policy and intervenes when necessary. This concept has appeared in imitation learning as well, for instance, in Human-Gated DAgger (HG-DAgger), where a human can take over if the learner’s confidence is low. In robotics, **Multiplicative Controller Fusion (MCF)** by Rana et al. [19] is notable: they trained an ensemble of navigation policies and a classical controller, fusing them such that the controller’s influence increased with policy uncertainty. MCF achieved safe sim-to-real transfer in navigation by falling back to the reliable controller in uncertain states. More recent work has pushed this logic toward runtime safety layers for embodied foundation models. **UNISafe** uses calibrated epistemic uncertainty in a world model to synthesize a latent-space safety filter that proactively avoids out-of-distribution failures [20]. **Modular Safety Guardrails** argue more broadly that foundation-model-enabled robots require explicit monitoring and intervention layers beyond end-to-end policies alone [21]. Similarly, our gating can be seen as a switching policy: learned policy when confident, classical planner when uncertain. Unlike MCF’s multiplicative blending, we use a simpler gating (on/off) which is sufficient in our manipulation tasks and easier to analyze. In terms of theoretical grounding, our method connects to **chance-constrained control**: we essentially ensure the probability of unsafe outcomes remains below a set threshold (if the model is calibrated). This idea has been explored in motion planning under uncertainty and in frameworks like *Safe-IL* that guarantee safety with high probability during learning. Our specific contribution is to study how post hoc calibration changes the practical operating point of a long-horizon VLA supervisor, including not only success and collisions but also intervention frequency and runtime burden.

### 2.4. Domain Shift Evaluation and Structured Randomization

Bridging the sim-to-real gap is a longstanding goal in robotics. **Domain randomization (DR)** is a widely adopted strategy wherein one trains a policy or vision model on a distribution of simulated environments with varied textures, lighting, etc., so that the real world appears as just another variation. Pioneered by Tobin et al. [22] for object detection, DR has enabled zero-shot transfer of grasping and navigation policies to real robots by randomizing visual and physical parameters during training. Over time, researchers realized that naive randomization (e.g., purely random textures or object placements) might not efficiently teach the model; instead, preserving *structure* in the randomization yields better generalization. **Structured Domain Randomization (SDR)** proposed by Prakash et al. [23] generates synthetic scenes that respect the semantic context (e.g., cars on roads, not floating). They showed that SDR outperformed unstructured randomization on transferring object detectors to KITTI, especially for scenarios requiring context (occluded, small objects).

In our work, we employ SDR principles to create training and evaluation scenarios: for example, in the drawer task, distractor objects are only placed on surfaces or inside containers (never floating arbitrarily), and lighting variations follow plausible indoor patterns. We find that training with structured random variations yields a policy that is more robust and whose uncertainty estimates are more meaningful. We systematically vary one factor at a time in evaluation to quantify its impact on success (e.g., increase motion blur magnitude or dim lights). This approach complements other sim-to-real techniques like domain adaptation, but since we do not fine-tune on real data, we lean entirely on randomization for visual generalization. Our contribution here is not a new randomization method per se, but the design of a comprehensive evaluation protocol for domain shift relevant to manipulation (covering lighting, material, occlusion, sensor noise, etc., as detailed in Section 5.2). We hope this can serve as a blueprint for future sim-to-real safety evaluations.

## 3. Preliminaries and Problem Setup

### 3.1. Vision–Language–Action Policy Model

We consider a pre-trained VLA policy $\pi_{\theta }\left(o,w\right)$ which takes the robot’s observation *o* (e.g., one or more camera images) and a textual task instruction *w* as inputs, and outputs a robot action *a*. The action *a* may be a continuous six-DoF end-effector motion command (possibly discretized via tokens) along with discrete gripper actions (open/close) and an optional task termination flag. We make minimal assumptions regarding $\pi_{\theta }$’s architecture; in experiments, we use OpenVLA, which encodes (o,w) via a vision–language transformer and decodes actions as a sequence of discrete tokens, but our method is equally applicable to other architectures (e.g., latent diffusion policies or a behavior-cloned policy with continuous Gaussian outputs).

We assume access to $\pi_{\theta }$’s *confidence* or *uncertainty* estimate about its chosen action. In practice, this can be derived in multiple ways:

- If $\pi_{\theta }$ outputs a full probability distribution over actions (as in a discrete token model), we can extract measures like maximum softmax probability, entropy, or the margin between top actions. For sequence decoders, sequence-level likelihood or average token probability can serve as a confidence score.
- We can augment $\pi_{\theta }$ with a scalar *self-estimated success probability* q(o,w) alongside *a*. This score can be learned with an auxiliary success head or by retrospective episode-level success classification.
- We can use an **ensemble** of *M* policies ${\left\{\pi_{\theta_{i}}\right\}}_{i=1}^{M}$ (or an efficient approximation like a dropout ensemble) and quantify epistemic uncertainty by the disagreement among $\left\{\pi_{\theta_{i}}\left(o,w\right)\right\}$. For instance, the variance in predicted action vectors or the mutual information in the ensemble’s output distribution indicates uncertainty.

Let u(o,w) denote the chosen uncertainty metric, where higher *u* means the policy is less sure about its action. In our formulation, *u* will be mapped to an estimate of *failure probability* (through calibration).

We treat the VLA policy as a black box in terms of how it was trained. In experiments, we fine-tune the base model on our tasks using a modest number of synthetic demonstrations (to ensure it can perform the task sequence), but crucially, we do not modify the model to improve its safety or uncertainty awareness. This separation of concerns allows our safety gating to be applied post hoc to any learned policy.

### 3.2. Uncertainty Quantification and Calibration

For gating decisions, we separate uncertainty into:

- **Epistemic uncertainty** $u_{e}$ arising from model ignorance or distributional shift (e.g., a novel situation not seen in training). High $u_{e}$ typically implies the model’s prediction may be arbitrary and risky.
- **Aleatoric uncertainty** $u_{a}$ arising from inherent stochasticity in the environment or observations (e.g., sensor noise, or the task itself being partially observable). High $u_{a}$ might imply the model’s best possible prediction still has a certain probability of error that cannot be reduced by more training.

**Figure 1:**
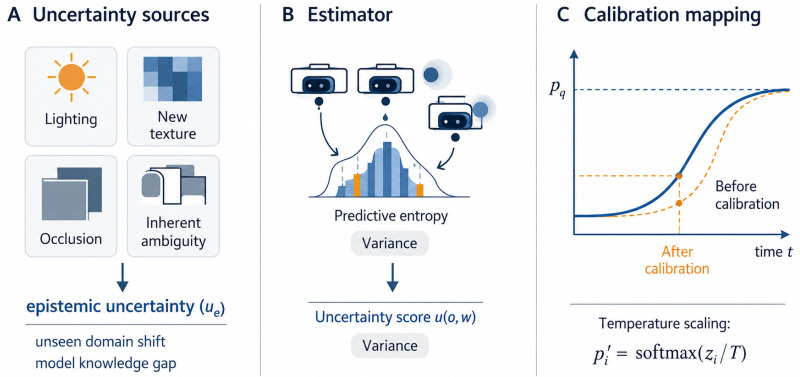
Uncertainty and calibration schematic. (**A**) Epistemic vs. aleatoric uncertainty sources under domain shift and sensing ambiguity. (**B**) Example uncertainty estimator (ensemble/dropout) producing a predictive distribution and summary statistics. (**C**) Calibration mapping (temperature scaling) converting uncertainty to calibrated success probability ${\hat{p}}_{\mathrm{succ}}\left(o,w\right)$ or calibrated risk $r_{t}$ (schematic; no real data).

In practice, ensemble variance primarily captures epistemic uncertainty, whereas predictive entropy may reflect a mix of epistemic and aleatoric effects. Our gating rule does not require a strict decomposition. Still, the *fallback planner* (Section 4.2) is best viewed as a mitigation for epistemic risk: it is reliable in known conditions but cannot fully resolve fundamentally unpredictable situations. Figure 1 summarizes the uncertainty sources, estimator output, and calibration mapping used by the proposed gate.

For clarity, we log and analyze the uncertainty channels separately. The confidence channel $1-c_{t}$ is derived from the selected action or token sequence probability and is most sensitive to local action ambiguity. The entropy channel $h_{t}$ measures dispersion of the predictive distribution and, therefore, reacts to ambiguous visual or language-conditioned action choices. The ensemble/action-variance channel $v_{t}$ measures disagreement among stochastic or independently fine-tuned policy variants and is intended to capture epistemic uncertainty. The deployed scalar $u_{t}$ is a composite score calibrated to empirical task failure; therefore, any improvement from gating should not be attributed to a single channel without a component diagnostic. Section 6.3 reports such a diagnostic and shows which signals actually carry failure-predictive information in the cleaned package.

#### Calibration

We define $p_{\mathrm{succ}}\left(o,w\right)=P\left(\mathrm{task}\;\mathrm{success}|o,w,\mathrm{use}\;\mathrm{learned}\;\mathrm{policy}\right)$ as the true probability that the robot will complete the task from state *o* given instruction *w* if it continues with the learned policy (and does not intervene). The policy’s raw confidence (or an uncertainty score *u*) is generally a poor proxy for this probability. Therefore, we learn a calibration function $f_{c}$ such that ${\hat{p}}_{\mathrm{succ}}\left(o,w\right)=f_{c}\left(u\left(o,w\right)\right)$ approximates $p_{\mathrm{succ}}\left(o,w\right)$.

We adopt temperature scaling for simplicity. If the policy provides a success probability estimate q(o,w), we define the logit z(o,w)

$$z\left(o,w\right)=\log\frac{q(o,w)}{1-q(o,w)},$$

and find T>0, minimizing the negative log-likelihood on a calibration dataset $D_{\mathrm{cal}}$:

(1) $$\begin{array}{cc} T=\arg\min_{T^{\prime }>0}\sum_{\left(o,w,y\right)\in D_{\mathrm{cal}}}\; & -[y\log\sigma \;\left(\frac{z(o,w)}{T^{\prime }}\right)\\ \; & \;+\left(1-y\right)\log\;\left(1-\sigma \;\left(\frac{z(o,w)}{T^{\prime }}\right)\right)], \end{array}$$

where y∈{0,1} indicates success and σ(·) is the logistic function. The calibrated success probability for the observation–instruction pair (o,w) is then

$${\hat{p}}_{\mathrm{succ}}\left(o,w\right)=\sigma \;\left(\frac{z(o,w)}{T}\right).$$

If T>1, predictions were over-confident and are made “softer”. For ensemble-based uncertainty, one can similarly calibrate by fitting a mapping from ensemble variance to actual error frequency (e.g., via isotonic regression, which we compare in experiments). We evaluate calibration quality via **Expected Calibration Error (ECE)** and reliability diagrams, ensuring our gating decisions are based on well-calibrated probabilities.

### 3.3. Problem Definition

We now formalize the manipulation tasks and safety requirements. Each task is a sequential decision problem where the robot must achieve a goal *G* (e.g., object *A* in bin *B*, drawer closed) from an initial state, following a possibly multi-step instruction *w* (or a series of instructions). The state evolves in discrete time steps t=0,1,… until success or failure. The VLA policy $\pi_{\theta }$ provides actions $a_{t}$ based on the current observation $o_{t}$. We assume that if left unchecked, the policy may incur a **safety violation**: an event such as a collision, a joint limit breach, dropping an object, or any defined unsafe condition. We denote by V the set of states where a violation has occurred (e.g., robot in contact with environment in an unintended way). We seek to ensure with high probability that V is never reached.

At the same time, the policy may also **fail** to complete the task (without an obvious safety event) due to, say, getting stuck or making a wrong move that precludes progress. We denote task success as reaching goal *G* and failure as not reaching *G* within a time budget or a certain number of attempts (this includes safety terminations). Domain shift is modeled as the test environment having a different distribution of parameters (lighting, textures, etc.) than the policy was tuned for. Our evaluation protocol (Section 5.2) defines various **domain shift levels** along multiple axes.

Under this setup, our **objectives** are to:

1. Maximize task success rate under domain shifts.
2. Minimize safety violations (ideally zero collisions or dangerous events).
3. Minimize performance degradation on nominal (in-distribution) scenarios.
4. Characterize robustness as performance degrades with shift severity, and test whether calibrated gating narrows that degradation.

The safety gating system introduced next is designed to intervene only when necessary, to satisfy (2) without unduly hurting (1) and (3).

## 4. Method: Uncertainty-Calibrated Safety Gating

**Figure 2:**
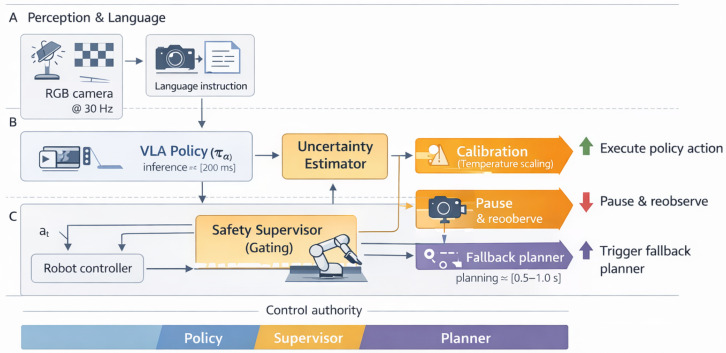
System architecture of the proposed wrapper. (**A**) Perception and language inputs, including the RGB camera stream and language instruction, are provided to the VLA policy. (**B**) The VLA policy $\pi_{\theta }$ generates the candidate action $a_{t}$, while the uncertainty estimator computes $u_{t}$ and the calibration module maps $u_{t}\mapsto r_{t}$ using temperature scaling. (**C**) The safety supervisor gates control authority by executing the policy action, pausing to reobserve, or triggering the fallback planner.

An overview of our approach is illustrated in Figure 2 (conceptual diagram). The learned policy $\pi_{\theta }$ operates in parallel with an uncertainty estimator and a safety logic module that decides whether to trust $\pi_{\theta }$’s action or override it. We first describe the gating mechanism and then the design of fallback behaviors, followed by a theoretical discussion on risk reduction.

### 4.1. Risk-Aware Gating Mechanism

Our gating policy is defined by a risk metric $r_{t}$ computed at each time step *t* based on the current observation $o_{t}$, instruction *w*, and the VLA policy’s behavior. We define $r_{t}$ as the calibrated probability of failure if the learned policy continues without intervention:

(2) $$r_{t}:={\hat{P}}_{f}\left(o_{t},w\right)=1-{\hat{p}}_{\mathrm{succ}}\left(o_{t},w\right),$$

where ${\hat{p}}_{\mathrm{succ}}\left(o_{t},w\right)$ is the calibrated success probability discussed earlier. Intuitively, $r_{t}$ estimates “how risky is it to trust the policy at this point?”

We introduce two threshold parameters $0<\delta_{\mathrm{low}}<\delta_{\mathrm{high}}<1$ to implement a simple hysteresis, enabling three regimes:

- **Safe to proceed ($r_{t}<\delta_{\mathrm{low}}$):** Execute the action $a_{t}$ normally.
- **Borderline ($\delta_{\mathrm{low}}\leq r_{t}<\delta_{\mathrm{high}}$):** Invoke a *cautious slowdown or re-observation* strategy. We implement this as pausing and feeding the next camera frame (from a slightly different viewpoint) to $\pi_{\theta }$ to see if $r_{t}$ changes.
- **Unsafe to proceed ($r_{t}\geq \delta_{\mathrm{high}}$):** Disengage the learned policy and hand control to the *fallback controller* (Section 4.2).

**Figure 3:**
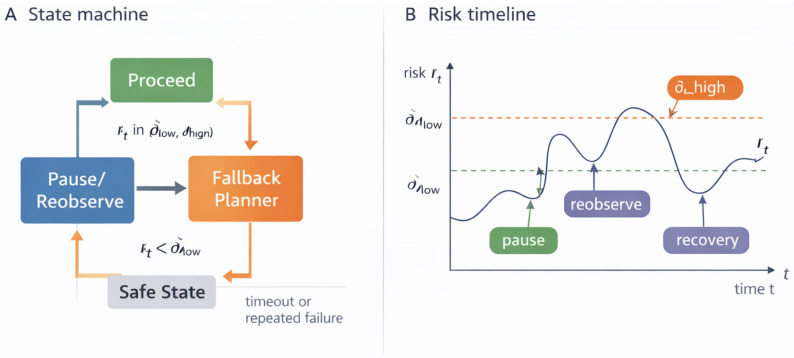
Safety gating logic. (**A**) State machine with hysteresis thresholds $\delta_{\mathrm{low}}$ and $\delta_{\mathrm{high}}$ governing transitions between proceed, pause/reobserve, fallback planning, and a safe state (schematic). (**B**) Example risk trajectory $r_{t}$ illustrating threshold crossings and intervention events (schematic; no numeric values).

This gating logic is summarized in Algorithm 1. Figure 3 visualizes the corresponding state machine and a representative risk timeline. In our experiments, we use $\delta_{\mathrm{low}}=0.2$ and $\delta_{\mathrm{high}}=0.5$. These values should be interpreted as an operating point, not as universal constants: $\delta_{\mathrm{high}}$ controls the intervention–miss trade-off and $\delta_{\mathrm{low}}$ controls the size of the pause/reobserve band. The values are selected on a held-out validation set rather than on the final test table: failures often produce calibrated risk spikes near or above 0.5, while a lower pause boundary of 0.2 avoids converting ordinary visual ambiguity into excessive replanning. Section 6.2 reports a retrospective threshold-sensitivity analysis over shifted logs and shows how the selected point compares with more conservative and more aggressive alternatives.
**Algorithm 1:** Uncertainty-Guided Safety Gating (at time *t*)
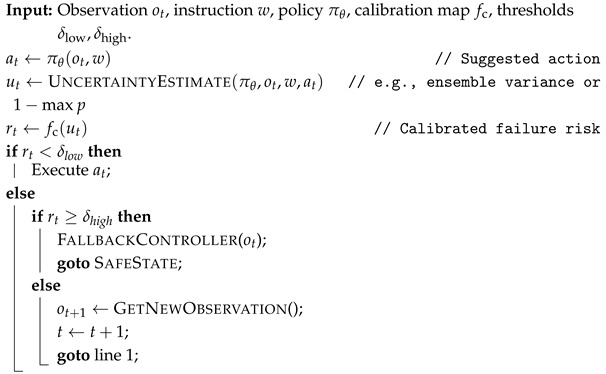


### 4.2. Fallback Controller and Recovery Behaviors

As the fallback, we use a classical motion planning pipeline based on **MoveIt 2**. The fallback controller has a library of high-level recovery behaviors, each consisting of a motion plan that is computed on-the-fly given the scene state:

- **Retreat to Safe Pose:** Move to a known safe configuration (e.g., home pose).
- **Re-orient for Visibility:** Move camera/end-effector to improve scene visibility, then hand control back to $\pi_{\theta }$.
- **Complete Task via Planner:** For certain phases, plan and execute safe motions to complete subtasks (e.g., place object into a known bin pose).
- **Recovery from Failure:** Attempt graceful recovery (e.g., re-grasp) when feasible.

The fallback planner is not assumed to be a formally verified, always-successful controller. It is a structured recovery mechanism with known limits: planning can time out, the planner can operate with an incomplete collision scene, and a geometrically valid trajectory can still be suboptimal for task progress when perception is stale or small clutter is not modeled. In this implementation, fallback execution is bounded by the MoveIt 2 planning time limit and by safe-pose/reobserve recovery behaviors; episodes are still counted as failures if collision thresholds or task-completion criteria are violated. Therefore, we evaluate fallback behavior empirically in Section 6.4 and avoid claiming that fallback eliminates all risk.

### 4.3. Theoretical Rationale for Risk Reduction

In an idealized scenario, if our failure probability estimates were perfectly accurate and if the fallback action were guaranteed safe, the gating rule could be interpreted as a chance-constrained controller. For example, setting $\delta_{\mathrm{high}}=0.1$ would mean that the system proceeds only when the estimated one-step continuation risk is at most 10%. Over a horizon of *H* independent decision opportunities, a conservative union-bound argument would upper-bound the probability of any continuation failure by 0.1H. The real system does not satisfy the assumptions needed for a formal guarantee: calibration error is nonzero, contacts are history-dependent, and fallback is empirical rather than certified. We use this rationale only to motivate risk-aware switching; Section 6 reports observed reliability, collisions, and residual failures.

## 5. Experimental Setup

### 5.1. Simulator, Robot, and System Integration

All primary experiments are conducted in **NVIDIA Isaac Sim 5.0** running on an Ubuntu 22.04 workstation. We use an NVIDIA RTX 4080 GPU (16 GB VRAM) for both simulation and neural network inference. The physics timestep is 0.005 s and rendering at 30 Hz unless otherwise noted. Our robot is the **Franka Emika Panda** 7-DoF arm, chosen for its wide adoption and availability of a high-fidelity Isaac Sim model. The Panda is equipped with a parallel-jaw gripper. We attach an RGB-D camera to the robot’s wrist (eye-in-hand view) and also have a static overhead RGB camera. Most experiments use only RGB inputs (from one or two cameras of 640×480 resolution) unless depth is explicitly part of a scenario. The observations $o_{t}$ fed to the policy consist of these camera images (we also provide the textual instruction *w* each episode).

The VLA policy backbone is **OpenVLA (7B)**, which we run using mixed-precision inference on the GPU. OpenVLA was originally trained on diverse manipulation data, and we found it had some zero-shot ability for our tasks (especially for high-level language understanding). However, to adapt to our exact action space and scene, we perform a light fine-tuning: we script 50 demonstration episodes for Task A and 70 for Task B in simulation (using the ground-truth simulator state to generate successful trajectories) and fine-tune OpenVLA on this using behavior cloning for 5 epochs (approximately 5k gradient steps, taking ∼2 hours on our GPU). This yields a specialized policy for each task, which still uses the pre-trained visual-language encoders of OpenVLA and, thus, generalizes better than training from scratch. We emphasize that our safety gating method is *not* tied to OpenVLA specifics and indeed we also test with the **Octo** model (93 M parameters, more lightweight) to ensure generality. At runtime, the policy inference takes ∼200 ms per step (dominated by the transformer forward pass), which is acceptable for our tasks, as motions are slow and episodic.

For uncertainty estimation, our default uses an **ensemble of size 5**. We create five slight fine-tuning variants of the policy (by training with different random seeds on the demonstration set) and run all five in parallel at each decision (this is feasible because the policy is relatively small in the Octo case; for OpenVLA 7B, we instead use MC dropout with 20 stochastic forward passes due to memory constraints). The ensemble variance in predicted action tokens and the entropy of the mean distribution are combined to form $u_{t}$. In an ablation, we also try a simpler approach: using the model’s output probability for the chosen action (or the cumulative log-probability of the output sequence) as a confidence score.

The safety gating logic and fallback controller are implemented in Python as part of an ROS 2 node that interfaces with Isaac Sim. When gating triggers a fallback, we pause the physics, invoke MoveIt 2 (running the OMPL planner) to get a trajectory, and then execute it by sending joint commands to the simulated robot. The overhead of planning is about 0.5–1.0 s for typical motions (20–30 waypoint trajectory). We find this integration stable; the ROS 2 Bridge in Isaac Sim allows for using real MoveIt without domain adjustments.

### 5.2. Tasks and Domain Shift Scenarios

**Figure 4:**
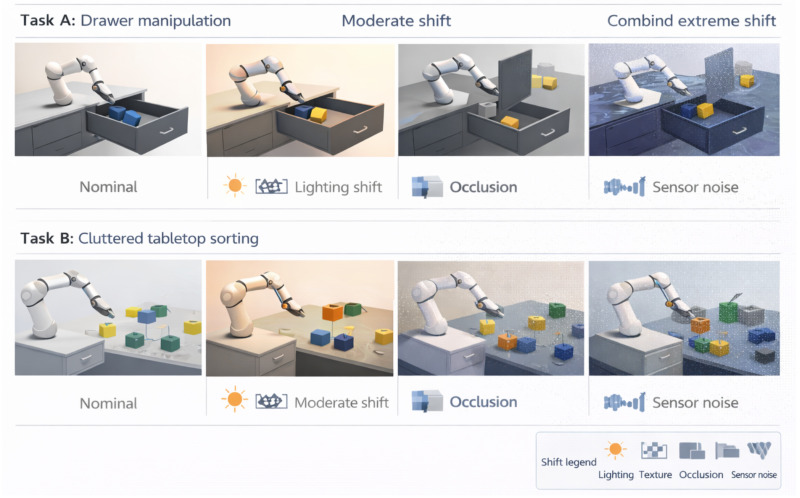
Long-horizon manipulation tasks and illustrative domain shifts (stylized, non-photorealistic). **Top row**: Task A (drawer manipulation) under nominal conditions, a moderate shift, and a combined extreme shift. **Bottom row**: Task B (cluttered tabletop sorting) under the same shift severities. Icons indicate representative perturbations (lighting, texture, occlusion, sensor noise).

We design two primary tasks:

(A) **Drawer-Object Task:** The robot is instructed, for example, “Open the drawer, pick up the red block inside, and place it in the green bin. Then close the drawer.” This requires a sequence: approach and pull drawer handle, reach inside to grasp the target, place it, and then push drawer closed. The scene (see Figure 4 Task A) has a cabinet with a sliding drawer, a colored block inside, and a bin on the table.
(B) **Cluttered Pick-And-Sort Task:** Instruction example: “Find the blue cylinder among the objects on the table and put it on the left tray. Put any green object on the right tray.” This scenario (Figure 4 Task B) has 5–7 random objects (various shapes) scattered. The robot may need to move obstructing objects to access the target. It also may need to re-grasp objects if initially picked awkwardly. This tests more open-world behavior and multi-step planning not strictly sequential (it can pick up distractors first).

Each task is long-horizon: (A) typically 4 distinct sub-goals, (B) potentially 3–5 pick–place cycles. Success only occurs if all parts are done correctly. We deliberately include stages where the policy could easily err under confusion (e.g., in (A), it might knock the drawer or miss the block if vision is off; in (B), it might grasp the wrong object).

We define **domain shift axes** and create multiple levels for each:

- **Lighting:** We vary illumination intensity (0.5×, 1×, 2× baseline), color temperature (cool vs. warm light), and direction (moving the light source angle). Level 0 has normal lighting, level 1 has moderate dimming or color shift, and level 2 has dim + colored lighting and heavy shadows.
- **Materials/Textures:** We randomize object and furniture textures. In high shift, the textures include reflective or transparent materials (glossy drawer front, shiny objects), which can confuse vision. Level 0 uses uniform matte colors (as in training demos), level 1 uses random realistic textures (wood grain, plastic patterns) for each object, and level 2 includes one or more objects with reflective metal or transparent plastic shaders.
- **Occlusion/Clutter:** We vary the presence of additional clutter objects and their positions to occlude targets. In (A), level 1 might put an extra item in the drawer partially covering the block, while level 2 puts two items there, plus one hanging partially out of the drawer (increasing occlusion and requiring moving them). In (B), we vary the density: level 0 has 5 well-separated objects, level 1 has 7 objects with some touching or stacked, and level 2 has 10 objects filling the workspace (significant occlusions and the robot must move things out of the way).
- **Sensor Noise and Degradation:** We simulate camera issues: Gaussian blur, motion blur (during robot movement), Gaussian noise on pixel values, and JPEG compression artifacts. Level 1 might add slight blur (σ=1 pixel) and mild noise, while level 2 adds strong blur (σ=3 pixels), low resolution (downsample 2× and upsample), and random dropout of one camera at times. We also simulate an extreme case where the depth sensor fails (for runs where the policy might rely on depth).
- **Distractors and Background:** Additional irrelevant objects or background changes. E.g., in (A), we place random unrelated objects on the table or floor; at high levels, we even place an unrelated moving object (like a toy car passing by) to test attention. Background change includes floor texture pattern changes and wall color shifts.

We designate a certain combination as the **training domain**: for this, we randomize lighting in [0.8×,1.2×], with a limited set of 5 textures per object (all matte), up to 1 extra distractor, and normal sensor quality. The policy is fine-tuned on that domain using demonstrations. Then, we evaluate on progressively shifted domains: we either change one factor at a time to a high level to isolate its effect, or in **combined shifts**, we sample all factors at once from their level ranges to mimic a challenging real deployment scenario.

We compile, on average, 100 test episodes for each condition (ensuring variability by re-sampling random seeds and positions). This yields a comprehensive dataset for evaluation, on which we can compute success rates, etc., with confidence intervals.

### 5.3. Baseline Methods for Comparison

We compare the following controllers:

- **VLA Policy (No Gating):** The raw learned policy $\pi_{\theta }$ executes actions continuously until task completion or a failure halts it. This represents the baseline performance and risk without any uncertainty intervention.
- **Uncalibrated Thresholding:** To test the importance of calibration, we use the same ensemble uncertainty measure $u_{t}$ but simply set a threshold on an ad hoc scale (tuned on nominal data). If $u_{t}$ exceeds this threshold, we trigger the fallback immediately. Essentially, this is gating without proper calibration of what *u* means. We tune the threshold to get roughly the same number of interventions on nominal runs as our calibrated method for fairness.
- **Calibrated Gating (Ours):** The full method described, with temperature scaling calibration (learned on a small validation set drawn from moderate shifts), $\delta_{\mathrm{low}},\delta_{\mathrm{high}}$ as described, and our multi-tier actions (pause or fallback).
- **Fallback-on-Failure Oracle:** This baseline lets the VLA policy run freely, *but* if a failure (collision or obvious miss) happens, it then triggers the MoveIt fallback to recover and attempt to finish the task. This is akin to a “post hoc rescue” strategy. It is not an ideal baseline for safety since a collision has occurred, but it indicates what happens if we only react after failures rather than before. It uses the same fallback behaviors as ours, just triggered later.

Additionally, for ablations, we consider:

- **Ours w/ Dropout vs. Ensemble:** Replacing the ensemble with MC dropout to gauge any difference in uncertainty quality or compute overhead.
- **Ours w/ Different Calibration:** Using isotonic regression (non-parametric) instead of temperature scaling, and also a variant with no calibration (just use raw ensemble variance normalized to [0,1]) to see the impact on gating performance.
- **Gating Variants:** (i) Single threshold (no intermediate pause, just a single cutoff to either go or fallback) and (ii) a more aggressive gating that always fallbacks on any threshold exceedance without pause.
- **Fallback Behavior Set:** We try a minimal set (only retreat-to-safe and abort vs. our full set that also does task-completion where possible) to see if completing via fallback yields better success or if it is risky.
- **Randomization Strategy:** We compare training the policy with structured DR (our approach) vs. unstructured DR (completely random textures/lighting with no regard to context) vs. no DR (train in nominal only) to see how these affect baseline and gated performance under shift.

### 5.4. Metrics and Evaluation Protocol

We measure:

- **Success Rate (%):** The fraction of episodes where the task is fully completed. Multi-step tasks count as success only if all sub-goals are done in order.
- **Safety Violations:** We log any collision events, whether they are transient or cause failure. We report the average number of collisions per episode and the percentage of episodes with any collision. We also monitor any high force/torque event (like exceeding a force threshold if the robot pushes on an object too hard).
- **Intervention Rate:** For gating methods, how often (in how many episodes, and how many times within an episode) the fallback was triggered. A lower number indicates the learned policy was trusted more often; however, too low might mean we missed catching some risky cases (so we examine together with violation count).
- **Episode Time and Efficiency:** We record time to completion and the number of actions executed. Gating and fallback often increase time; we quantify the overhead. If an episode fails or is aborted, we take the maximum time as a penalty in the average.
- **Calibration Metrics:** We compute ECE (10-bin) on the predictions of success probability versus actual outcomes for the policy on a variety of scenarios. We also generate reliability diagrams for the calibration analysis.The NLL (negative log-likelihood) of the success/failure outcomes given predicted probabilities is also reported.
- **Robustness vs. Severity Curves:** We report success as a function of structured shift severity and use these curves to quantify how rapidly each method degrades outside nominal conditions.
- **Threshold Sensitivity:** We retrospectively sweep calibrated risk thresholds on shifted ungated logs. This does not substitute for a full closed-loop rerun at every threshold, but it quantifies how many failed episodes would have been flagged and how much successful behavior would have been interrupted.
- **Failure-Mode and Signal Diagnostics:** We join episode, step, intervention, and contact logs to identify whether residual failures occur in proceed, pause/reobserve, or fallback states, and we compute individual uncertainty-signal discriminative power for final episode failure.

For each metric, we report the mean over 3 random seeds (each seed generates a set of test episodes with different random initializations) and 95% confidence intervals (for success and violation rates, these are Wilson score intervals for binomial proportions). We also run pairwise significance tests in the analysis pipeline (chi-square for success rates and *t*-tests for time-related metrics, with correction where appropriate), but because the manuscript tables aggregate across conditions and are intended to foreground effect sizes, intervals, and operating-point differences, we summarize those results in the text rather than using table-level significance markers.

All training and test parameters (random seeds, hyperparameters like δ values, temperature *T*, etc.) are listed in the reproducibility checklist (Section 11).

## 6. Results

### 6.1. Main Benchmark Results

**Figure 5:**
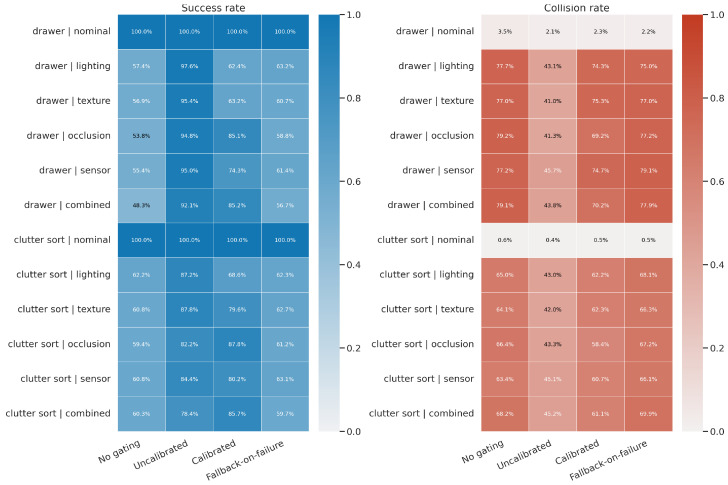
Cleaned benchmark summary regenerated from the benchmark package. **Left**: success-rate heatmap. **Right**: episode-level collision-rate heatmap. Nominal rows pool severity 0 episodes; shifted rows pool severities 1–3 within each shift axis.

The cleaned benchmark package provides a benchmark summary with a single aggregation rule: the nominal row pools severity 0 episodes across shift axes, while each shifted row pools severities 1–3 within a given axis. Table 1 and Figure 5 report the resulting task-completion and collision statistics.

Three findings are directly supported by the cleaned benchmark. First, calibrated gating consistently improves robustness relative to the ungated VLA under every shifted condition. Averaged over all ten shifted task/axis combinations, success increases from 57.5% to 77.2% (+19.7 points). The largest gains occur on *drawer/combined* (48.3% → 85.2%) and *clutter sort/occlusion* (59.4% → 87.8%). Second, the safety benefit of calibrated gating is measurable but modest: the mean shifted episode-level collision rate falls from 71.7% to 66.9%, with the clearest reductions on drawer occlusion and drawer combined. Third, the uncalibrated threshold baseline is the strongest raw performer on this cleaned benchmark, reaching 89.5% mean shifted success and 43.4% mean shifted collision rate, but it does so by intervening much more aggressively than the calibrated policy. Taken together, the results support a Pareto-style interpretation rather than a single best-method claim: the ungated VLA, calibrated supervisor, and aggressive threshold controller occupy meaningfully different points on the robustness, safety, and intervention frontier. Table 2 makes this operating-point comparison explicit by placing success, collision, interventions, and runtime in one compact view.

Nominal performance is saturated at 100% success for all methods, so the discriminative evidence comes almost entirely from shifted conditions. This makes the benchmark package particularly useful for studying the trade-off between robustness, safety, and intervention aggressiveness outside the training distribution. For deployment, the key point is that intervention count is not merely a bookkeeping variable: each intervention adds latency, planner dependence, and operator-facing complexity, so a lower-intervention operating point can be preferable even when it is not the raw best point on a benchmark frontier.

**Figure 6:**
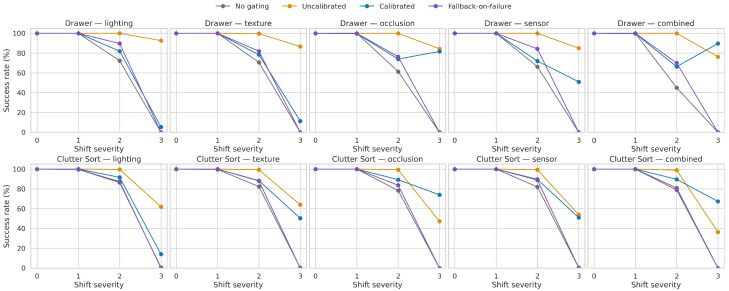
Success rate versus shift severity for each task and domain-shift axis, regenerated from the cleaned logs. Calibrated gating degrades more gracefully than no gating across all axes, but the uncalibrated threshold baseline remains the strongest raw performer on most severity 3 conditions.

Figure 6 sharpens this picture at the severity level. Across nearly all axes, the ungated policy collapses as severity approaches level 3. Calibrated gating preserves a much shallower degradation curve, especially for occlusion and combined shifts. However, the same figure also shows that a highly interventionist threshold policy can outperform calibrated gating on raw terminal success, suggesting that calibration quality alone is not sufficient; the downstream risk-to-action mapping remains a central design variable.

### 6.2. Threshold Sensitivity

Table 3 reports a retrospective sensitivity sweep of the high-risk threshold on shifted ungated logs. For each episode, we computed the maximum calibrated failure risk observed before termination. A threshold is counted as covering a failed episode if any step in that failed episode exceeded the threshold, and as interrupting a successful episode if any step in that successful episode exceeded the threshold. This analysis does not claim the same terminal success that a closed-loop rerun would produce; it isolates the operating-point question of how sensitive the trigger is to the numerical threshold.

The selected $\delta_{\mathrm{high}}=0.5$ is, therefore, a middle operating point: it retains high failed-episode coverage (97.1%) while reducing successful-episode interruption from 65.3% at τ=0.4 to 34.3%. A more conservative τ=0.6 would interrupt fewer successful episodes but would miss more failures (failed-episode coverage falls to 85.9%). The lower threshold controls the pause band rather than fallback triggering: holding $\delta_{\mathrm{high}}=0.5$, the mean number of pause-eligible steps per shifted ungated episode would be 15.5 for $\delta_{\mathrm{low}}=0.1$, 12.1 for the selected 0.2, and 7.1 for 0.3. These results support the manuscript’s interpretation that the gate is a tunable supervisor rather than a threshold-free safety proof.

### 6.3. Calibration and Uncertainty Quality

**Figure 7:**
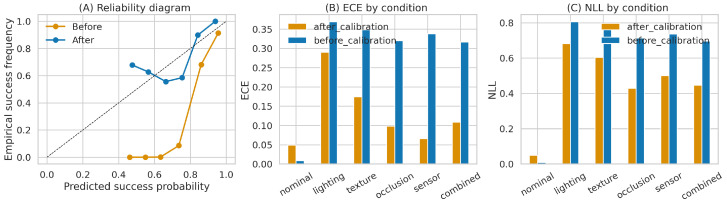
Calibration analysis regenerated from the cleaned benchmark package. (**A**) Reliability diagram before and after calibration. (**B**) Expected calibration error by condition. (**C**) Negative log-likelihood by condition. Calibration substantially improves reliability on all shifted conditions, even though the nominal row slightly worsens because it is already near-perfect before recalibration.

The strongest quantitative evidence in favor of the proposed method is predictive reliability under shift. Aggregate ECE drops from 0.3025 before calibration to 0.1003 after calibration, while aggregate NLL drops from 0.6703 to 0.4853. Table 4 gives the condition-level values, and Figure 7 visualizes the corresponding reliability diagram, ECE, and NLL results. The gains are consistent across all shifted conditions, with the largest ECE improvements under sensor shift (0.338 → 0.066), occlusion (0.320 → 0.098), and combined shift (0.317 → 0.109). The only regime in which calibration is worse is the nominal setting, where the uncalibrated predictor is already nearly perfect (ECE 0.009 vs. 0.049 after calibration). This indicates that the calibration machinery is primarily valuable as an out-of-distribution reliability correction rather than an in-distribution optimization. Importantly, the calibration set used moderate randomization only; the table, therefore, reports transfer to stronger and combined shifts rather than recalibration on every test condition.

Table 5 reports the package’s ablation slice across backbone and calibration variants. This table is intentionally interpreted as a local ablation slice, not as a contradiction of the aggregate calibration results in Table 4. On that split, OpenVLA with calibrated gating achieves the highest success rate (89.0%) and best NLL (0.492), while removing calibration from the same backbone reduces success to 77.0% and raises intervention frequency from 25.8 to 37.6 interventions per episode. The *OpenVLA* + *no calibration* row has a lower slice-level ECE because ECE is sensitive to bin occupancy, class balance, and the distribution of states produced by the intervention policy; it should, therefore, not be read as evidence that calibration is globally worse. The more stable reading is based on the joint metrics: calibration improves likelihood quality and yields the strongest success/intervention trade-off on this ablation split, while Table 4 provides the broader condition-wise reliability evidence.

Table 6 separates the logged uncertainty channels by their ability to rank final episode failure on shifted ungated steps. In this package, the confidence/entropy channels dominate the failure-predictive signal, whereas the ensemble/action-variance scalar is much weaker by itself. This does not mean epistemic uncertainty is irrelevant in general; it means that, for the cleaned benchmark and the present implementation, the observed gains should be attributed mainly to calibrating the policy-confidence/entropy signal and then acting on it through the supervisor. This clarification prevents over-claiming that every mentioned uncertainty component contributed equally.

### 6.4. Intervention Behavior and Safety Characterization

**Figure 8:**
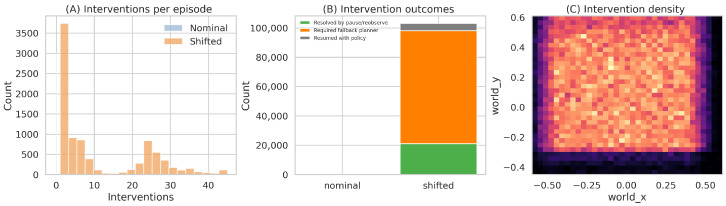
Intervention analysis regenerated from the cleaned logs. Shifted conditions induce a long-tailed intervention-count distribution. Most calibrated interventions ultimately route to the fallback planner, while a smaller but non-trivial fraction is resolved by pause/reobserve.

The intervention logs explain why calibrated gating occupies a different operating point from the uncalibrated baseline. Averaged over shifted episodes, calibrated gating uses 11.5 interventions per episode versus 21.6 for the uncalibrated threshold baseline. Within calibrated gating, 75.8% of interventions escalate to the fallback planner, 19.7% are resolved by pause/reobserve, and 4.5% resume nominal policy execution without planner takeover. Thus, the pause tier is used meaningfully, but planner escalation remains the dominant mechanism in the cleaned runs. Figure 8 summarizes the intervention-count distribution and the resulting intervention outcomes.

**Figure 9:**
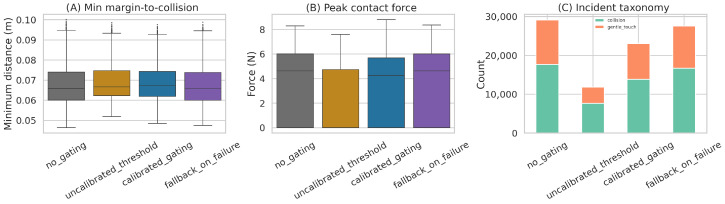
Safety characterization from the cleaned benchmark package. The calibrated supervisor slightly improves contact statistics relative to no gating, but the strongest raw safety profile on this benchmark is obtained by the more aggressive uncalibrated threshold baseline.

The contact statistics are similarly mixed. Relative to no gating, calibrated gating slightly lowers mean peak contact force (4.69 N → 4.36 N) and reduces the total number of contact events. However, it does *not* achieve near-zero collisions on the cleaned benchmark. The uncalibrated threshold baseline yields the fewest contact events and the lowest mean peak contact force (2.69 N), but this comes with substantially higher supervisory overhead and longer wall-clock runtime (8.70 s per shifted episode vs. 5.07 s for calibrated gating and 1.53 s for no gating). Thus, calibrated gating adds 3.54 s over no gating on average in shifted episodes, while the more aggressive threshold baseline adds 7.17 s. This cumulative overhead is material for long-horizon manipulation because repeated pauses and fallback calls compound across sub-goals. Figure 9 summarizes the corresponding safety and contact statistics.

Table 7 summarizes the residual failure diagnostics for calibrated gating under shifted conditions. Most calibrated interventions route to the fallback planner, but residual collision contacts are not confined to raw policy execution: 63.7% occur while the system is in the pause/reobserve state and 28.1% occur during fallback. The dominant phases are placing in the bin, grasping the block, and routing to the tray. This pattern indicates that remaining failures are mixed: some are perception/uncertainty errors, some are action-execution errors near contact-rich subtasks, and some are fallback/planner limitations in cluttered or partially observed scenes. Therefore, the fallback planner improves recovery but is not equivalent to a certified safe controller.

### 6.5. Scope of Evidence

The current benchmark package does not contain dedicated cleaned results for ensemble vs. dropout uncertainty, structured vs. unstructured randomization, or physical-robot evaluation. Accordingly, all quantitative claims in this paper are limited to the controlled simulation benchmark. No claim of real-world safety certification, physical-robot transfer, or deployment readiness is made. The simulated shifts deliberately stress appearance, occlusion, sensing, and combined visual perturbations, but they should be interpreted as a controlled stress-test of uncertainty supervision rather than as evidence that the system closes the physical sim-to-real gap. Any statements about real-robot transfer or randomization strategy remain qualitative until matching hardware logs or summary tables are added.

## 7. Discussion

The cleaned benchmark supports a nuanced conclusion. Calibrated gating is a substantially better-behaved supervisor than an ungated VLA under domain shift, and it dramatically improves predictive calibration, but it does not dominate a more aggressive uncalibrated threshold baseline on raw benchmark success or collision rate. The scientific contribution is, therefore, the measured operating-point characterization itself: calibrated supervision trades some raw terminal performance for fewer interventions, better probability semantics, and lower runtime burden than aggressive thresholding.

This distinction matters scientifically. The available evidence suggests that there are at least two separate problems in uncertainty-aware robot supervision: (i) estimating failure risk accurately and (ii) mapping that risk to the right intervention policy. The calibration results show clear progress on the first problem. The benchmark comparison shows that the second problem remains open: an aggressive threshold controller can achieve stronger raw outcomes by intervening much more often, even when its uncertainty estimates are less principled. In other words, uncertainty quality and intervention policy quality should be evaluated jointly but not conflated.

### 7.1. What the Cleaned Benchmark Supports Strongly

- **Calibration under shift works:** Aggregate ECE and NLL improve substantially after calibration, especially under sensor, occlusion, and combined shifts, even though the calibration set is smaller and milder than the strongest test shifts.
- **Calibrated supervision is better than no supervision:** Across all shifted conditions, calibrated gating improves task success over the ungated VLA and modestly reduces collision rates.
- **Intervention burden is a first-class metric:** The calibrated supervisor achieves its gains with roughly half as many interventions as the uncalibrated threshold baseline, which is important for runtime efficiency and for preserving learned behavior when the policy is still reliable. The overhead analysis shows that this difference translates directly into wall-clock cost.

### 7.2. What the Cleaned Benchmark Does Not Support

- **Near-zero collision claims:** Those claims are not consistent with the cleaned benchmark exports.
- **Universal dominance over threshold baselines:** On this package, uncalibrated thresholding is stronger on raw success and collision metrics.
- **Quantitative sim-to-real claims:** The current benchmark package does not include corresponding real-robot logs, so the study should be read as controlled simulation evidence rather than deployment validation.

### 7.3. Simulation-to-Deployment Gap and Deployment Risk Analysis

Although the benchmark deliberately introduces structured lighting, texture, occlusion, sensor, and combined shifts, it should not be interpreted as closing the sim-to-real gap. The present evidence evaluates whether calibrated uncertainty can improve runtime supervision under controlled distribution shift, not whether the complete system is certified for physical deployment. Real deployment would introduce additional error sources that are only partially represented here, including camera exposure and lens artifacts, imperfect object-pose estimation, contact and friction mismatch, actuator latency, gripper compliance, incomplete collision scenes, and human-proximity constraints. These factors could affect both the VLA policy and the fallback planner, and they may also change the calibration of the risk score itself.

Table 8 summarizes how the main deployment gaps relate to the current benchmark and what validation would be needed before physical deployment claims could be made. This analysis is intentionally conservative: the current study provides simulation evidence for a risk-supervision mechanism, while deployment readiness remains conditional on staged physical validation.

A practical validation pathway would, therefore, proceed in three stages. First, real-camera replay or hardware-in-the-loop evaluation should test whether the calibrated risk scores remain reliable under physical sensor streams while the robot is not executing risky motions. Second, constrained tabletop trials should run the same ungated, calibrated-gated, and aggressive-threshold operating points at reduced speed with external workspace guarding. Third, full closed-loop physical manipulation should compare success, contact/collision rate, intervention burden, latency, calibration error, and fallback failures under matched real-world shifts. This staged pathway makes clear that task success alone would be insufficient; the relevant deployment evidence is the joint profile of reliability, safety, calibration, intervention efficiency, and runtime behavior.

### 7.4. Implications

An appropriate framing is an *accuracy–intervention trade-off frontier*. In that framing, the contribution is that calibration produces a more interpretable and less intervention-heavy supervisor that still markedly outperforms the ungated baseline under shift. A strong next step is to improve the policy that converts calibrated risk into pause, fallback, or safe-stop actions so that the calibrated method can move closer to the raw benchmark frontier achieved by the aggressive threshold controller.

## 8. Limitations and Future Work

- **Simulation-only validation and deployment gap:** All quantitative results are from Isaac Sim. The current package does not include matched physical-robot runs, hardware-in-the-loop timing tests, or real-camera replay studies. Therefore, the results validate a controlled simulation operating point, not physical deployment readiness.
- **Scope of Shifts:** We focused on visual domain shifts and simple physical shifts, not extensive dynamics shifts (mass/friction).
- **Policy Model Dependence:** We assumed a usable uncertainty signal; some policies may require an auxiliary predictor.
- **Calibration Data Requirement:** We used a few hundred runs for calibration; self-calibration is a promising direction.
- **Fallback Limitations:** MoveIt 2 handles structured motions, but the fallback controller is not formally verified and can still produce suboptimal or collision-prone behavior when the scene estimate is incomplete, clutter is unmodeled, or recovery requires higher-level task reasoning.
- **Theoretical Guarantees:** We provide rationale but no formal proof; formal certification would require bounded calibration error, verified fallback actions, and explicit treatment of temporal dependence across long-horizon decisions.
- **Generalization to other modalities:** Extending beyond vision–language is plausible but may need different uncertainty signals.

Future work could include:

- **Adaptive thresholding:** Dynamic δ based on context or learning a gating policy that optimizes safety/efficiency.
- **Learning when to ask for help:** Trigger human assistance rather than only autonomous fallback.
- **Scaling to fleet learning:** Share intervention data across robots.
- **Improving VLA robustness:** Use intervention states as additional fine-tuning data.
- **Human factors:** Study how mode switching is perceived and how to communicate it clearly.

## 9. Ethical and Safety Considerations

This work is motivated by safety in robotic AI deployments. By adding an uncertainty-aware guard, we aim to reduce the risk of physical harm or damage from errant policies. The proposed supervisor should be understood as an additional runtime assurance layer, not as a substitute for certified low-level safety mechanisms, workspace guarding, or human oversight.

Considerations include:

- **Residual Risk:** Risk is reduced but not eliminated; conservative thresholds and physical guards are advised.
- **Misuse or Overtrust:** Operators may over-rely on gating; this is not a license to operate recklessly.
- **Transparency:** Mode switching should be transparent to nearby humans; cues may be useful.
- **Data bias:** If training/calibration misses certain scenarios, uncertainty may be less reliable there.
- **Compute and Energy:** Large models and ensembles are energy-intensive; compression and efficient uncertainty approximations are important.
- **Reproducibility and auditability:** Independent validation of safety claims benefits from sharable evaluation artifacts, careful reporting, and access-managed data release when full public distribution is not feasible.

## 10. Conclusions

We presented an uncertainty-calibrated safety-gating framework for VLA-based robotic manipulation under domain shift and evaluated it with a cleaned benchmark package spanning structured visual shifts. The evidence supports a nuanced but still meaningful conclusion: calibrated gating substantially improves shifted robustness relative to an ungated VLA and strongly improves predictive reliability, but it does not dominate an aggressive uncalibrated threshold baseline on raw success or collision metrics. The central contribution is, therefore, not a universal-best controller claim, but a clear empirical characterization of how calibration changes the operating point of a runtime supervisor for embodied manipulation.

Specifically, the revised evidence shows where calibrated supervision sits on the reliability–intervention frontier: it improves success and calibration over no supervision, uses roughly half as many shifted-episode interventions as aggressive thresholding, and incurs less runtime overhead than the raw-best threshold controller. This evidence-grounded interpretation highlights the importance of calibration under distribution shift and exposes the open systems question of how best to act on calibrated uncertainty. Future work should pair the current calibration machinery with more effective risk-to-action policies and extend the benchmark through staged deployment validation: real-camera replay or hardware-in-the-loop calibration tests, constrained low-speed physical manipulation, and finally full closed-loop robot trials under matched shifts. Within the present scope, the manuscript provides a simulation-bounded account of trustworthy VLA supervision under structured domain shift; it does not claim real-world safety certification, sim-to-real transfer, or physical deployment validation.

### Reproducibility Statement

We provide detailed descriptions of the simulation environments, model architectures, evaluation protocol, and training procedures used to obtain the reported results. In addition, Section 11 enumerates the hyperparameters, hardware, and implementation details needed to reproduce the benchmark package.

## 11. Reproducibility Checklist

- **Hardware Used:** Simulations ran on a single desktop with AMD Ryzen 5950X CPU @ 3.4 GHz, 64 GB RAM, and NVIDIA RTX 4080 16 GB GPU.
- **Software Versions:** Isaac Sim 5.0 (binary release), which corresponds to internal version 5.0. Python 3.8, PyTorch 2.0, CUDA 11.7. ROS 2 Foxy for MoveIt 2 (MoveIt 2 version 2.2.0). OpenVLA model weights from an arXiv v3 release (Sept. 2024). The operating system was Ubuntu 22.04 with NVIDIA driver 525.
- **Simulation Environment:** Scenes were built in USD. Domain randomization was implemented via Isaac Replicator: we wrote Python scripts to randomize lights (intensity uniformly in [0.5,2.0], color temperature random from 3000 K to 7000 K), apply textures (from a set of 20 texture images including wood, marble, plastic patterns—drawn uniformly, but structured by applying wood textures to furniture, etc.), add clutter objects (random selection of 5 object meshes from the Isaac Sim assets library, placed at random free spots on the table or in the drawer as specified by scenario). Occlusions in Task A: we placed up to 2 extra blocks inside the drawer at random positions. Clutter in Task B: object spawn positions were jittered normally around predetermined spots; at high clutter, we also allowed stacking by dropping objects from a slight height so they could settle in piles.
- **Demonstrations and Training:** 50 demonstrations for Task A and 70 for Task B were generated by a scripted policy (using ground-truth poses). Each demo was ∼100–150 timesteps. Fine-tuning hyperparameters: learning rate $10^{-5}$, batch size 8, Adam optimizer, 5 epochs (with early stopping if validation loss stopped improving for 1 epoch). We used 10% of demos as validation for early stop.
- **OpenVLA Integration:** We used an OpenVLA HuggingFace checkpoint (7B parameters). It requires ∼14 GB GPU memory for full FP16 inference. Images were resized to 224×224 and encoded via the model’s ViT; language was tokenized via the model’s tokenizer. OpenVLA output action tokens which we decoded to continuous commands (trained with a 256-token discretization per joint delta). We used the provided codebook to map tokens to delta end-effector motions. We aligned units so that one token step corresponded to ∼1 cm move, etc. Octo (93 M) was used similarly following its observation normalization.
- **Ensemble:** We fine-tuned 5 separate variants by training only the final layers on our demo data with different random seeds. For MC dropout, we inserted dropout layers (drop probability 0.1) in the feedforward networks of each transformer block during inference and did 20 stochastic forward passes.
- **Uncertainty to Risk Mapping:** We created a calibration dataset of 200 episodes (100 per task, moderate randomization) where we ran the policy and recorded outcome (success/failure) and the policy’s raw confidence features (ensemble mean max-probability, as well as variance). We then applied temperature scaling (Equation (1)). The optimal temperatures were T≈2.5 for Task A and T≈2.2 for Task B.
- **Threshold Selection:** $\delta_{\mathrm{high}}$ and $\delta_{\mathrm{low}}$ were chosen based on calibrated risk values on a separate 50-episode set. We observed that in collision cases, ${\hat{P}}_{f}$ often spiked near or above 0.5 before the event, so we set $\delta_{\mathrm{high}}=0.5$. We set $\delta_{\mathrm{low}}=0.2$ to avoid excessive pausing. The revised manuscript added a retrospective sweep (Table 3), showing that increasing $\delta_{\mathrm{high}}$ reduced interruption but missed more failures, whereas lowering it increased intervention burden.
- **Motion Planner:** MoveIt 2 was configured with Panda’s URDF. Planning time limit: 2 s per plan. We used RRT-Connect. The collision scene was updated with known static meshes (table, drawer) and known object primitives (approximated as boxes or cylinders). We did not model small clutter in the collision scene for planning; therefore, fallback motions bias upward first to avoid unknown clutter.
- **Metrics Calculation:** Collision was flagged if any contact force >5 N. We instrumented Isaac Sim’s contact report for robot links vs. environment. Success was checked by verifying the object was in the bin (position check) and the drawer was closed (drawer joint value).
- **Statistical Analysis:** We ran each condition with 3 random seeds (object placements, etc.). That gave roughly 300 episodes per condition. Confidence intervals in the table used Wilson score for proportions. Significance was assessed by pairwise chi-square tests on successes, with Bonferroni correction across conditions.
- **Artifact Availability:** The manuscript source, bibliography, and figure assets required to rebuild the submitted article PDF are included in the submission package. Additional benchmark artifacts and underlying evaluation data are available from the corresponding authors upon reasonable request.

## Figures and Tables

**Table 1:** Cleaned benchmark results from the benchmark package. Success is reported as percentage with 95% confidence interval; collision is the episode-level collision rate in percent.

Task	Condition	No Gating		Uncalibrated Threshold		Calibrated Gating		Fallback-on-Failure	
		Success	Collision	Success	Collision	Success	Collision	Success	Collision
Drawer retrieval	Nominal	100.0 (99.7–100.0)	3.5	100.0 (99.7–100.0)	2.1	100.0 (99.7–100.0)	2.3	100.0 (99.7–100.0)	2.2
	Lighting	57.4 (54.2–60.6)	77.7	97.6 (96.3–98.4)	43.1	62.4 (59.2–65.5)	74.3	63.2 (60.0–66.3)	75.0
	Texture	56.9 (53.6–60.1)	77.0	95.4 (93.9–96.6)	41.0	63.2 (60.0–66.3)	75.3	60.7 (57.4–63.8)	77.0
	Occlusion	53.8 (50.5–57.0)	79.2	94.8 (93.1–96.1)	41.3	85.1 (82.6–87.3)	69.2	58.8 (55.5–61.9)	77.2
	Sensor	55.4 (52.2–58.7)	77.2	95.0 (93.4–96.2)	45.7	74.3 (71.4–77.1)	74.7	61.4 (58.2–64.6)	79.1
	Combined	48.3 (45.1–51.6)	79.1	92.1 (90.2–93.7)	43.8	85.2 (82.8–87.4)	70.2	56.7 (53.4–59.9)	77.9
Clutter sort	Nominal	100.0 (99.7–100.0)	0.6	100.0 (99.7–100.0)	0.4	100.0 (99.7–100.0)	0.5	100.0 (99.7–100.0)	0.5
	Lighting	62.2 (59.0–65.3)	65.0	87.2 (84.9–89.2)	43.0	68.6 (65.4–71.5)	62.2	62.3 (59.1–65.4)	68.1
	Texture	60.8 (57.5–63.9)	64.1	87.8 (85.5–89.8)	42.0	79.6 (76.8–82.1)	62.3	62.7 (59.5–65.8)	66.3
	Occlusion	59.4 (56.2–62.6)	66.4	82.2 (79.6–84.6)	43.3	87.8 (85.5–89.8)	58.4	61.2 (58.0–64.4)	67.2
	Sensor	60.8 (57.5–63.9)	63.4	84.4 (81.9–86.7)	45.1	80.2 (77.5–82.7)	60.7	63.1 (59.9–66.2)	66.1
	Combined	60.3 (57.1–63.5)	68.2	78.4 (75.6–81.0)	45.2	85.7 (83.2–87.8)	61.1	59.7 (56.4–62.8)	69.9

**Table 2:** Compact shifted-condition operating-point summary. Success, collision, and intervention counts are averaged over the shifted rows in Table 1; wall-clock time is averaged over shifted episode logs. The table highlights that calibrated gating is not the raw-best controller, but rather a lower-intervention calibrated point between no supervision and aggressive thresholding.

Controller	Shifted Success (%) ↑	Collision Rate (%) ↓	Interventions/ Episode ↓	Wall Time/ Episode (s) ↓	Interpretation
No gating	57.5	71.7	0.0	1.53	Fast baseline; poor shifted reliability
Uncalibrated threshold	**89.5**	**43.4**	21.6	8.70	Raw-best but highly interventionist
Calibrated gating	77.2	66.9	11.5	5.07	Calibrated lower-intervention supervisor
Fallback-on-failure	61.0	72.4	1.5	2.04	Late recovery after failures emerge

*Note*: ↑ indicates that higher values are better, whereas ↓ indicates that lower values are better. Bold values indicate the best value in each metric column.

**Table 3:** Retrospective sensitivity of the high-risk trigger on shifted ungated episodes. τ denotes a candidate value for $\delta_{\mathrm{high}}$. Coverage is the percentage of failed episodes with at least one step above τ; interruption is the percentage of successful episodes with at least one step above τ; trigger steps are averaged over all shifted ungated episodes.

τ	Failed Episodes Covered (%)	Successful Episodes Interrupted (%)	Trigger Steps/Episode
0.30	100.0	71.2	21.6
0.40	99.9	65.3	18.7
0.50	97.1	34.3	14.5
0.60	85.9	11.9	11.2
0.70	78.4	0.1	6.5

**Table 4:** Condition-wise calibration transfer. Values are computed from the cleaned step logs. Lower ECE and NLL are better.

Condition	ECE Before	ECE After	NLL Before	NLL After
Nominal	0.009	0.049	0.009	0.050
Lighting	0.370	0.290	0.806	0.681
Texture	0.349	0.175	0.782	0.604
Occlusion	0.320	0.098	0.714	0.428
Sensor	0.338	0.066	0.736	0.500
Combined	0.317	0.109	0.697	0.446

**Table 5:** Backbone and calibration ablation from the cleaned results package.

Variant	ECE ↓	NLL ↓	Success (%) ↑	Interventions ↓
OpenVLA + calibrated gating	0.269	**0.492**	**89.0**	**25.8**
Octo + calibrated gating	0.306	0.589	85.0	27.7
OpenVLA + no calibration	**0.158**	0.500	77.0	37.6

*Note*: ↑ indicates that higher values are better, whereas ↓ indicates that lower values are better. Bold values indicate the best value in each metric column.

**Table 6:** Component-level uncertainty diagnostics on shifted ungated logs. AUROC and average precision (AP) evaluate step-level scores against final episode failure labels; higher is better. Mean scores are shown separately for steps from failed and successful episodes.

Signal	AUROC	AP	Mean Score, Failed Episodes	Mean Score, Successful Episodes
1—raw policy confidence	0.946	0.959	0.291	0.080
Predictive entropy	0.946	0.959	0.573	0.259
Ensemble/action variance	0.609	0.603	0.00317	0.00300
Calibrated failure risk	0.946	0.959	0.646	0.298

**Table 7:** Residual intervention and failure diagnostics for calibrated gating on shifted episodes. Percentages are computed from the cleaned intervention and contact logs.

Diagnostic	Category	Share (%)
Intervention disposition	Fallback planner	75.8
	Pause/reobserve resolved	19.7
	Resume policy without planner	4.5
Collision contacts by gating state	Pause/reobserve	63.7
	Fallback	28.1
	Proceed	8.1
Top collision phases	Place in bin	29.7
	Grasp block	22.0
	Route to tray	16.8

**Table 8:** Simulation-to-deployment gap analysis. The current benchmark covers selected visual and supervisory stressors, but physical deployment would require additional staged validation.

Deployment Gap	Current Benchmark Coverage	Residual Risk and Required Future Validation
Visual appearance shift	Lighting, texture, occlusion, sensor degradation, and combined visual shifts are explicitly varied.	Real cameras add exposure changes, lens distortion, motion artifacts, auto-white-balance effects, and unmodeled background clutter; validation should include real-camera replay and hardware-in-the-loop perception tests under matched perturbations.
Scene geometry and perception	Clutter and occlusion are simulated, and fallback planning uses an estimated collision scene.	Physical object poses, mesh approximations, and collision-scene updates may be incomplete or delayed; validation should use estimated, not ground-truth, poses in constrained tabletop trials.
Contact dynamics and grasp physics	Contact events and peak forces are logged in simulation.	Drawer friction, gripper compliance, object slip, impacts, and deformable contacts may differ from Isaac Sim dynamics; validation should include physical grasp, drawer, and contact-rich manipulation trials with force/contact monitoring.
Runtime latency and synchronization	Wall-clock overhead for pause and fallback actions is reported in the cleaned runs.	Real ROS communication, perception pipelines, controllers, and actuator response may change closed-loop behavior; validation should measure inference, planning, communication, and actuation delay in hardware-in-the-loop tests.
Fallback-planner safety	Residual contacts during fallback are explicitly reported rather than assumed away.	MoveIt trajectories may be unsafe if the scene model is stale, clutter is unmodeled, or recovery requires higher-level task reasoning; validation should include fallback-only and gated-fallback trials at reduced speed in a guarded workspace.
Operational and human safety	Ethical and safety considerations are discussed, but no human-proximity experiment is performed.	Deployment requires workspace guarding, emergency stops, speed limits, operator procedures, and safety certification; validation should begin with fenced, low-speed trials before any human-proximity operation.

## Data Availability

The data supporting the findings of this study are available from the corresponding authors upon reasonable request.
